# Identifying hypermethylated CpG islands using a quantile regression model

**DOI:** 10.1186/1471-2105-12-54

**Published:** 2011-02-15

**Authors:** Shuying Sun, Zhengyi Chen, Pearlly S Yan, Yi-Wen Huang, Tim HM Huang, Shili Lin

**Affiliations:** 1 Case Comprehensive Cancer Center, Case Western Reserve University, Cleveland, Ohio, USA; 2 Department of Epidemiology and Biostatistics, Case Western Reserve University, Cleveland, Ohio, USA; 3 Department of Statistics, Case Western Reserve University, Cleveland, Ohio, USA; 4 Human Cancer Genetics Program, The Ohio State University, Columbus, Ohio, USA; 5 Department of Statistics, The Ohio State University, Columbus, Ohio, USA

## Abstract

**Background:**

DNA methylation has been shown to play an important role in the silencing of tumor suppressor genes in various tumor types. In order to have a system-wide understanding of the methylation changes that occur in tumors, we have developed a differential methylation hybridization (DMH) protocol that can simultaneously assay the methylation status of all known CpG islands (CGIs) using microarray technologies. A large percentage of signals obtained from microarrays can be attributed to various measurable and unmeasurable confounding factors unrelated to the biological question at hand. In order to correct the bias due to noise, we first implemented a quantile regression model, with a quantile level equal to 75%, to identify hypermethylated CGIs in an earlier work. As a proof of concept, we applied this model to methylation microarray data generated from breast cancer cell lines. However, we were unsure whether 75% was the best quantile level for identifying hypermethylated CGIs. In this paper, we attempt to determine which quantile level should be used to identify hypermethylated CGIs and their associated genes.

**Results:**

We introduce three statistical measurements to compare the performance of the proposed quantile regression model at different quantile levels (95%, 90%, 85%, 80%, 75%, 70%, 65%, 60%), using known methylated genes and unmethylated housekeeping genes reported in breast cancer cell lines and ovarian cancer patients. Our results show that the quantile levels ranging from 80% to 90% are better at identifying known methylated and unmethylated genes.

**Conclusions:**

In this paper, we propose to use a quantile regression model to identify hypermethylated CGIs by incorporating probe effects to account for noise due to unmeasurable factors. Our model can efficiently identify hypermethylated CGIs in both breast and ovarian cancer data.

## Background

Epigenetic changes are one of the most common molecular modifications in cells [1-4]. Among different epigenetic changes, DNA methylation, the addition of a methyl group (CH_3_) to the 5's cytosine (C) at a CpG site, plays an important role in gene expression regulation, transposons silencing, and transcription factor binding inhibition [5-13]. Therefore, DNA methylation has significant implications in both normal biology and complex diseases, such as cancer. In fact, DNA methylation patterns change during tumor growth. These changes may include regional or genome-wide gain or loss of methylation [14]. The gain of methylation in cancer is called hypermethylation, that is, there are more methylation signals in cancerous cells than in normal cells. On the other hand, the loss of methylation in cancer is called hypomethylation, that is, there are fewer methylation signals in cancerous cells than in normal cells. Numerous studies have reported that DNA hypermethylation may cause tumor suppressor gene silencing [15,16]. These abnormal DNA methylations usually occur at CGIs, genomic regions rich in CpG sites.

In order to gain an understanding of how genome-wide (especially CGI) methylation changes affect tumor growth, numerous microarray protocols have been developed to simultaneously assay the methylation status of all or partial regions in the whole genome. Most of these microarray protocols are developed based upon one of the following three methods of methylation-dependent treatment of DNA, each with its advantages and disadvantages [16]: (1) using methylation sensitive enzymes (such as HpaII and HinpI) to digest DNA, (2) using specific antibodies or methyl-binding proteins to obtain DNA fragments enriched with methylation signals, and (3) using sodium bisulfite to treat denatured DNA to convert unmethylated cytosine (C) to thymine (T).

In our group, the DMH protocol has been developed to simultaneously assay the methylation status of all known CGIs [17,18] using methylation sensitive enzymes HpaII and HinpI to digest DNA. As opposed to the earlier DMH protocol in which interrogated samples were hybridized onto CGI clone arrays with printed probes averaging 870 bp in length, the current DMH method assays the sample using CGI tiling arrays with much shorter probes (45 - 60 bp). Probe affinity, PCR effects, and many other measurable and unmeasurable confounding factors due to shorter probe length affect the observed methylation signals [19].

Previous DMH methylation microarray data analysis methods either propose an arbitrary log ratio cut off of 1.5 to detect differential methylation [20] or focus on modeling differential methylation at the probe level [21]. Due to the large impact of probe affinity and many confounding factors, a single high log ratio probe may not represent true biological signals. Furthermore, it can be misleading to select differentially methylated promoter regions based on independent probe signals. In addition, we are more interested in identifying hypermethylated regions as opposed to local changes detected by a difference in a single probe. To meet these biological interests and needs, we propose the use of a quantile regression model [22] in order to aggregate CGI probe signals for the identification of hypermethylated regions. Probe effects are directly incorporated into this proposed model. Genes with hypermethylated promoters can be easily selected according to their associated CGIs. The idea of using a quantile regression model to identify methylated CGIs was originally presented by our group as a poster at the 4th International Symposium on Bioinformatics Research and Applications. In that poster, we used a quantile regression model at 75% quantile. Although known methylated and unmethylated genes can be identified, we were unsure whether 75% would turn out to be the best quantile level.

In the following sections, we first give a brief introduction to our breast cancer cell line and ovarian cancer microarray data. We then explain how to use a quantile regression model to identify hypermethylated CGIs. Finally, we implement quantile regression models at different quantile levels and compare the performance of these models using three statistical measurements.

## Methods

We use two DMH microarray datasets generated from 40 breast cancer cell lines and 26 ovarian cancer patients. In particular, we use the 2-color 244K Agilent arrays hybridized with the test samples (e.g. the breast cancer cell lines) dye coupled with Cy5 (red) and a common normal reference dye coupled with Cy3 (green). The base two log ratio of red over green intensity, log_2_(Cy5/Cy3), is used as the observed methylation signal at each probe. For each array, dye effects are corrected using the standard within array LOESS normalization in the Bioconductor package "limma" [23]. We have explored several normalization methods and found that the standard LOESS normalization produces more consistent and reliable results than the others (data not shown).

In a common DMH experiment, it is desirable to identify CGIs that are hypermethylated in a large percentage of the total N samples (e.g., N cancer patients or N cancer cell lines). Therefore, one important goal of our DMH microarray study is to identify the CGIs that are commonly methylated in N samples (N = 40 for breast cancer data and N = 26 for ovarian cancer data). In order to control the noise due to measured and unmeasured factors such as GC content, scanner effects, and PCR effects that may affect the signals, we apply the following quantile regression model to each CGI:

$$Q_{\text{Ysp}}\;(\tau |sample_{s},\;probe_{p})=sample_{s}(\tau )+probe_{p}(\tau )$$

where Q_Ysp _(τ|*sample_s_, probe_p_*) is the τ-th conditional quantile of the observed probe log ratio of sample *s *at probe *p*, *sample_s _*represents the expected signal from the sample, and *probe_p _*denotes the probe effect. In the above quantile regression model, error terms are assumed to be independent and distribution-free. The regression coefficients, especially *sample_s _*and *probe_p_*, are estimated by formulating the quantile regression problem as a linear program [22]. In fact, both parameter estimation and inference are conducted using the R package "quantreg" [22]. An example of using this package to fit a quantile regression model for one CpG island has been provided (see Additional file 1).

In the above regression model, we let τ = 95%, 90%, 85%, 80%, 75%, 70%, 65% and 60%. We choose quantile levels over 50% because we are interested in identifying hypermethylated regions. In particular, for each sample (or cell line) effect from the quantile regression output, there is a p-value indicating whether a sample (or cell line) shows significant methylation signals at one particular CGI under the null hypothesis that *sample_s_(τ) = *0. The methylation level at each CGI is taken as the number of samples for which their associated p-values are less than a certain cutoff value p_0 _where we let p_0 _= 0.05, 0.04, 0.03, 0.02, and 0.01. For example, if a CGI has p-values less than 0.01 in 38 out of 40 breast cancer arrays, this indicates that this CGI may have very strong methylation signals across many samples.

In order to verify that our quantile regression model can identify the real methylation signals and to compare the results of our regression models at different quantile levels, we use known methylated and housekeeping genes as "positive" and "negative" controls respectively. In fact, 30 known hypermethylated genes [24-27] have been reported for breast cancer, and 32 known hypermethylated genes have been reported for ovarian cancer [28]. For both breast and ovarian cancer, 47 housekeeping genes [29] are selected as "negative" control (i.e., known unmethylated genes) due to their low methylation signals. Recall that the methylation score given to each CGI is the count of samples with p-value less than a cutoff point. At each p-value cutoff point p_0_, we have a methylation score for each CGI. Then, there are N_m _and N_HK _methylation scores with N_m _= 30 for breast cancer data, N_m _= 32 for ovarian cancer data, and N_HK _= 47 for unmethylated housekeeping genes. We choose these N_m _and N_HK _genes because each of them is associated with at least one CGI. Therefore, this paper will refer to these genes as N_m _methylated and N_HK _unmethylated CGIs.

In order to determine if known methylated and unmethylated CGIs are identified correctly, we use three different statistical measurements for known methylated and unmethylated CGIs. The first measurement is the area under a Receiver Operating Characteristic (ROC) curve, which we call "AUC" (Area Under Curve). A ROC is a graphical plot of the sensitivity vs. (1 - specificity) for a binary classifier system as its discrimination threshold varies. The ROC can also be represented equivalently by plotting true positive rates (TPR) vs. false positive rates (FPR). In this paper, the TPR is the fraction of known methylated CGIs that are correctly classified as methylated CGIs at a specific methylation score level C_0 _(0 ≤ C_0 _≤ N). The FPR is the fraction of known unmethylated housekeeping CGIs that are incorrectly classified as methylated CGIs at a specific methylation score level C_0_. The second measurement is the mean difference of methylation scores of two groups. We call this measurement mean.diff, that is ${\bar{x}}_{m}-{\bar{x}}_{HK}$, where ${\bar{x}}_{m}$ and ${\bar{x}}_{HK}$ are mean methylation scores for known methylated and unmethylated housekeeping CGIs. The third measurement is the mean difference of methylation scores of two groups of CGIs divided by their standard deviation. That is,$\frac{{\bar{x}}_{m}-{\bar{x}}_{HK}}{\sqrt{s_{m}^{2}/N_{m}^{}+s_{HK}^{2}/N_{HK}^{}}}$, where ${\bar{x}}_{m}$, ${\bar{x}}_{HK}$, $s_{m}^{2}$ and $s_{Hk}^{2}$ are the mean and variance of methylation scores for known methylated and housekeeping CGIs respectively, we call this measurement "T.stat". At each quantile level τ, the larger a statistical measurement is, the more evident that this quantile level is better at identifying methylated and unmethylated CGIs.

## Results

**Figure 1:**
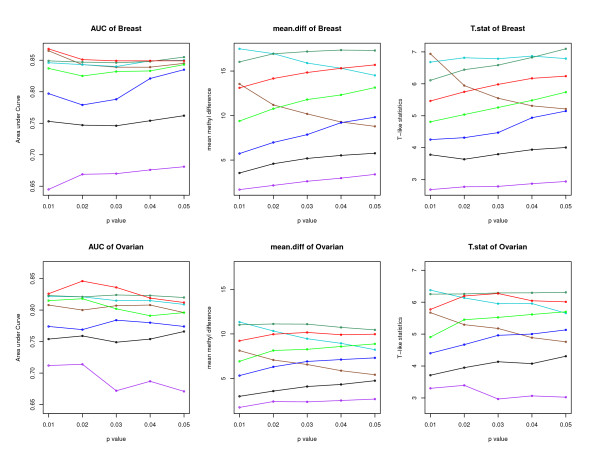
**Comparisons of quantile regression models at different quantile levels**. We compare results of different quantiles by studying their performances on identifying two different groups of CGIs (methylated and unmethylated). The legend is: "brown" for τ = 95%, "cyan" for τ = 90%, "dark green" for τ = 85%, "red" for τ = 80%, "green" for τ = 75%, "blue" for τ = 70%, "black" for τ = 65%, and "purple" for τ = 60%. The top panel contains three plots for breast cancer data while the bottom panel contains three plots for ovarian cancer data.

Using both breast and ovarian cancer data sets, we compare the performance of the proposed quantile regressions using three different measurements: AUC, mean.diff and T.stat. All comparison results are listed in Tables 1, 2, 3, 4, 5 and 6 with Tables 1, 2 and 3 for breast cancer data and Tables 4, 5 and 6 for ovarian cancer data. To have a clear view of these tables, we have plotted the summary result for each table in Figure 1, where the top three plots are for the three measurement results based on breast cancer data, and the bottom three plots are for the three measurements based on ovarian cancer data. For all three measurements, the larger a statistical measurement is, the better that a quantile regression model is at identifying the two different groups of CGIs (methylated and unmethylated). In Figure 1, we can see consistent patterns in all three measurements for both breast and ovarian cancer data. That is, 90% (cyan), the 85% (dark green), and 80% (red) are the top 3 lines and these three lines have relatively small variations across different p-values. Therefore, we can conclude that any τ between 80% and 90% could serve well to identify two different groups of CGIs (methylated and unmethylated). We recommend 85% for convenience.

**Table 1:** Breast cancer AUC measurement table

**τ**	**P < 0.01**	**p < 0.02**	**p < 0.03**	**p < 0.04**	**p < 0.05**
	
95%	**0.865**	0.843	0.839	0.839	0.845
90%	0.846	0.843	0.840	0.849	**0.850**
85%	0.849	0.847	0.846	0.848	**0.855**
80%	**0.868**	**0.851**	0.849	0.849	0.849
75%	0.837	0.825	0.832	0.833	0.843
70%	0.797	0.779	0.788	0.821	0.835
65%	0.753	0.747	0.746	0.754	0.762
60%	0.645	0.669	0.670	0.676	0.681

The first column contains the quantile levels. The second column contains a sub-table with each sub-column corresponding to the AUC measurement based on a specific p value and each sub-row corresponding to one quantile level. Bold numbers are the five largest AUC values in this table.

**Table 2:** Breast cancer mean.diff measurement table

**τ**	**P < 0.01**	**p < 0.02**	**p < 0.03**	**p < 0.04**	**p < 0.05**
	
95%	13.550	11.197	10.187	9.277	8.788
90%	**17.497**	**16.955**	15.899	15.312	14.515
85%	16.030	16.936	**17.191**	**17.357**	**17.309**
80%	13.114	14.165	14.837	15.312	15.694
75%	9.386	10.770	11.798	12.317	13.143
70%	5.732	6.981	7.860	9.204	9.818
65%	3.548	4.589	5.187	5.528	5.765
60%	1.682	2.156	2.618	2.972	3.395

The first column contains the quantile levels. The second column contains a sub-table with each sub-column corresponding to the mean.diff measurement based on a specific p value and each sub-row corresponding to one quantile level. Bold numbers are the five largest mean.diff values in this table.

**Table 3:** Breast cancer T.stat measurement table

**τ**	**P < 0.01**	**p < 0.02**	**p < 0.03**	**p < 0.04**	**p < 0.05**
	
95%	**6.939**	5.946	5.549	5.311	5.213
90%	6.680	**6.816**	6.786	**6.869**	6.790
85%	6.110	6.443	6.586	**6.826**	**7.097**
80%	5.463	5.749	5.982	6.172	6.241
75%	4.810	5.037	5.259	5.482	5.740
70%	4.254	4.313	4.472	4.938	5.147
65%	3.780	3.637	3.796	3.938	4.005
60%	2.686	2.774	2.787	2.872	2.941

The first column contains the quantile levels. The second column contains a sub-table with each sub-column corresponding to the T.stat measurement based on a specific p value and each sub-row corresponding to one quantile level. Bold numbers are the five largest T.stat values in this table.

**Table 4:** Ovarian cancer AUC measurement table

**τ**	**P < 0.01**	**p < 0.02**	**p < 0.03**	**p < 0.04**	**p < 0.05**
	
95%	0.808	0.800	0.807	0.808	0.796
90%	0.822	0.821	0.815	0.815	0.809
85%	0.823	0.821	**0.824**	**0.823**	0.820
80%	**0.826**	**0.846**	**0.836**	0.819	0.812
75%	0.815	0.818	0.802	0.791	0.796
70%	0.774	0.769	0.784	0.780	0.774
65%	0.754	0.759	0.749	0.754	0.766
60%	0.712	0.714	0.672	0.687	0.671

The first column contains the quantile levels. The second column contains a sub-table with each sub-column corresponding to the AUC measurement based on a specific p value and each sub-row corresponding to one quantile level. Bold numbers are the five largest AUC values in this table.

**Table 5:** Ovarian cancer mean.diff measurement table

**τ**	**P < 0.01**	**p < 0.02**	**p < 0.03**	**p < 0.04**	**p < 0.05**
	
95%	8.122	7.071	6.564	5.870	5.414
90%	**11.339**	10.331	9.459	8.942	8.229
85%	**11.022**	**11.119**	**11.099**	**10.734**	10.453
80%	9.226	9.966	10.166	9.909	9.969
75%	6.922	8.138	8.270	8.589	8.878
70%	5.320	6.301	6.908	7.119	7.310
65%	2.991	3.579	4.092	4.326	4.745
60%	1.745	2.407	2.369	2.521	2.682

The first column contains the quantile levels. The second column contains a sub-table with each sub-column corresponding to the mean.diff measurement based on a specific p value and each sub-row corresponding to one quantile level. Bold numbers are the five largest mean.diff values in this table.

**Table 6:** Ovarian cancer T.stat measurement table

**τ**	**P < 0.01**	**p < 0.02**	**p < 0.03**	**p < 0.04**	**p < 0.05**
	
95%	5.673	5.295	5.177	4.889	4.758
90%	**6.378**	6.134	5.953	5.958	5.656
85%	6.253	6.258	**6.290**	**6.293**	**6.308**
80%	5.772	6.194	**6.272**	6.044	6.010
75%	4.906	5.451	5.523	5.616	5.699
70%	4.400	4.671	4.962	5.002	5.132
65%	3.712	3.949	4.134	4.075	4.307
60%	3.302	3.395	2.967	3.065	3.024

The first column contains the quantile levels. The second column contains a sub-table with each sub-column corresponding to the T.stat measurement based on a specific p value and each sub-row corresponding to one quantile level. Bold numbers are the five largest T.stat values in this table.

In order to determine if our quantile regression model is better than other available methods, we compare our method with the previous one that uses a 1.5 cutoff value at a probe level [20] using our breast cancer data. A single probe with a large log ratio is not reliable, so we consider the following cases in each CGI: (1) at least 30% of probes with log ratios greater than 1.5, (2) at least 50% of probes with log ratios greater than 1.5, and (3) 100% probes (that is, all probes) with log ratios greater than 1.5. For the above three cases, the AUC is 0.51, 0.52, and 0.50. These small AUCs are mainly due to the fact that some methylated CGIs or genes do not necessarily have many probes with log ratios greater than 1.5. In fact, they are more likely to have several probes with relatively large but less than 1.5 log ratios. We see this pattern very often in our data. As for the first case, only 3 out of 30 known methylated genes and 4 out of 47 HK genes have at least one cell line with more than 30% probes that have log ratios greater than 1.5. As for the second case, only 2 out of 30 known methylated genes and 1 out of 47 HK genes have at least one cell line with more than 50% probes that have log ratios greater than 1.5. As for the third case, 0 out of 30 known methylated genes and 0 out of 47 HK genes have at least one cell line with 100% (i.e., all) probes that have log ratios larger than 1.5. Therefore, our quantile regression method is certainly much better than the one that uses 1.5 as a cutoff. In addition, the 1.5 cutoff method may work well for our previous version DMH protocol that has longer printed probes (about 870 bp). However, this arbitrary cutoff method does not work for the current protocol that uses much shorter probes (45 ~60 bp).

## Discussion

The three measurement plots of breast and ovarian cancer data have slightly different patterns. This may be due to the sample differences between the two datasets. Breast cancer data are generated from cell lines while ovarian cancer data are generated from patients. The breast cancer cell line samples are more homogeneous than ovarian patient samples. In addition, cancer cell lines appear to have more methylation than cancer patients. Furthermore, breast cancer data have 40 arrays and ovarian cancer data have 26 arrays. This sample size difference may also explain some inconsistencies between breast and ovarian cancer data at different quantile levels due to random variability.

We also observe that the results of the three proposed measurements show slightly inconsistent patterns. This may be due to the definition of the three measurements. AUC and T.stat both consider the variations of methylation scores. However, mean.diff only considers the difference of mean methylation scores between methylated CGIs and unmethylated housekeeping CGIs. Therefore, the result of AUC and T.stat may be more reliable.

## Conclusions

In this paper, we have proposed to use a quantile regression model to identify hypermethylated CGIs. In particular, we have incorporated probe effects to take into consideration the noises from unmeasurable factors. In order to find out at which quantile levels (95%, 90%, 85%, 80%, 75%, 70%, 65%, and 60%) the proposed quantile regression model is better at identifying known methylated and unmethylated CGIs, we have introduced three statistical measurements: AUC, mean.diff, and T.stat. These measurements show that the quantile level between 80% and 90% might serve well for identifying methylated and unmethylated CGIs. Although this paper has only demonstrated how to identify hypermethylated CGIs by setting quantiles larger than 50%, our quantile regression model can also be used to identify hypomethylated CGIs with quantiles smaller than 50%, if desired.

## Authors' contributions

SS and ZC developed and implemented the models, performed all statistical analyses, and drafted and revised the manuscript. PSY and YWH were involved in the data collection and helped in preparation of the manuscript. THMH oversaw the project and revised the manuscript. SL provided suggestions on the project and revised the manuscript. All authors have read and approved the final document.

## Supplementary Material

Additional file 1 **R code for fitting a quantile regression model**. This file gives an example of using the R package "quantreg" to fit a quantile regression model to identify methylation signals in one CpG island. Click here for file
