# Supplementary material for: Identifying hypermethylated CpG islands using a quantile regression model
Source: BMC Bioinformatics. 2011 Feb 15;12:54. doi: 10.1186/1471-2105-12-54 (PMC3051900; doi:10.1186/1471-2105-12-54)
Supplement: Additional file 1 — R code for fitting a quantile regression model. This file gives an example of using the R package "quantreg" to fit a quantile regression model to identify methylation signals in one CpG island. [file 1471-2105-12-54-S1.PDF]

Additional file 1:

```
#############################################################################################
#
# R code: Example of using quantile regression for methylation data at one CpG island
#
##############################################################################################


library("quantreg") # Load R package to run quantile regression

# Suppose the methylation data of one CpG island is saved in "M.norm.in.order", which is a
# matrix of  num.probe * num.array, where "num.probe" is the total number of probes in
# one CpG island and num.array is the total number of arrays or samples. For example,
# in one CpG island, num.probe =5 and num.array=40.

num.probe<-dim(M.norm.in.order)[1]
num.array<-dim(M.norm.in.order)[2]

CGI.log.ratio<-c(M.norm.in.order)  # This will be used as response variable.

# The following "array" and "probe" are fixed effects.
array<-as.factor(rep(1:num.array, each=num.probe) )
probe<-as.factor(rep(1:num.probe, time=num.array) )

# Fit a quantile regression model at 85% quantile for one CpG island
options(contrasts=c("contr.sum", "contr.poly") ) # Set up the contrast
fit<-rq(CGI.log.ratio ~ array + probe -1, tau =0.85, method="fn")

coefficients(fit)[1:num.array] # This will give the coefficients of all arrays or samples
coef(summary(fit, se="ker"))[1: num.array,4] # This will give p-value for all arrays or samples

# Note: the above setting is not proper for data sets that have replicates for each sample.
```
